# Relevance of light conditions for clinical *in situ* Raman spectroscopy

**DOI:** 10.3389/fonc.2026.1850523

**Published:** 2026-07-27

**Authors:** Sven Richter, Sotirios Kalousios, Roberta Galli, Jonathan Ziegler, Jakob Dremel, Achim Temme, Katrin Kirsche, Ilker Y. Eyüpoglu, Witold H. Polanski, Ortrud Uckermann

**Affiliations:** 1 Department of Neurosurgery, Faculty of Medicine and University Hospital Carl Gustav Carus, TUD Dresden University of Technology, Dresden, Germany; 2 Else Kröner-Fresenius Center for Digital Health, Faculty of Medicine, TUD Dresden University of Technology, Dresden, Germany; 3 Medical Physics and Biomedical Engineering, Faculty of Medicine, TUD Dresden University of Technology, Dresden, Germany; 4 Laboratory of Measurement and Sensor Systems, TUD Dresden University of Technology, Dresden, Germany; 5 German Cancer Consortium (DKTK), Dresden, Germany; German Cancer Research Center (DKFZ), Heidelberg, Germany; 6 National Center for Tumor Diseases Dresden, Dresden, Germany

**Keywords:** artifact, intraoperative, light, neuronavigation, Raman spectroscopy

## Abstract

**Background:**

Clinical Raman spectroscopy is a promising optical technology for intraoperative brain tumor delineation and diagnosis. As it exploits weak signals, illumination of the operating room (OR) and OR devices might impact the acquired spectra. The aim of this study was to investigate which optical artifacts can be tolerated during the intraoperative analysis of brain tumors *in situ* while still allowing the extraction of meaningful Raman spectra.

**Methods:**

Tissue samples of rat brain and human brain tumors were investigated in an experimental OR, and background signals and Raman spectroscopic datasets were acquired using a fiber-based system. The measurements were performed under various lighting conditions and OR equipment.

**Results:**

Ceiling light intensity increased the background, which could be prevented by manual coverage. Hence, the signal-to-noise-ratio of Raman spectra was not altered by ceiling lights. The intensity of the raw spectra of brain tissue and glioma was determined mainly by tissue autofluorescence and not by OR illumination, but it substantially affected the raw spectra of meningioma having low autofluorescence. Operating (OP) lights lead to saturation of the Raman camera. The exoscope, displays, and optical neuronavigation systems produced spectrally structured artifacts. Correct background subtraction was sufficient for artifact correction and obtaining valid Raman spectra.

**Conclusions:**

For clinical Raman spectroscopy, it is of utmost importance to be aware of optical disturbances. Intense illumination needs to be avoided but (spectrally structured) disturbances can be corrected by data preprocessing. Rather than external illumination, strong tissue autofluorescence might lead to impaired Raman spectral quality, making tumor tissue recognition difficult.

## Introduction

Raman spectroscopy is an analytical technique that operates purely optically: When a sample is illuminated, light-matter interactions, namely inelastic scattering, cause a shift in the wavelength of the light. The magnitude of this shift is specific to different molecular groups and can be visualized by spectrometers equipped with highly sensitive cameras in the form of a Raman spectrum. Raman spectroscopy probes the entire population of chemical groups in a sample and thus reveals its biochemical composition with high accuracy (1). Intensive basic research has shown that this technique is ideal for identifying tissue types when biological samples are being examined (2, 3). This includes the detection of malignant transformation in various types of cancers. Raman spectral differences between normal and tumor tissue have been reported for lung (4, 5), esophageal (6), cervical (7), colon (8–10), breast (11, 12), nasopharyngeal (13, 14) and brain cancer (15–18).

The fact that Raman spectroscopy operates optically, does not require labelling and can be used safely *in situ* makes it extremely attractive for clinical use, and several proof-of-concept applications highlight its usefulness for ex vivo and *in situ* intraoperative tumor detection. Breast cancer tumor margins were identified ex vivo using high wavenumber Raman spectroscopy (19). In neurosurgery, accurate determination of the tumor boundary and detection of tumor infiltration is of utmost importance, as the extent of resection and the residual tumor volume significantly affect clinical outcome (20–23). For successful maximal and safe tumor resection, tumor delineation is crucial. Therefore, new strategies for intraoperative tumor delineation are continuously being investigated and developed. Here, fiber-based Raman systems might provide surgical guidance (24). They have been successfully applied in rodent brain tumor models (25, 26) and were first tested in glioblastoma patients in 2015 (27). Raman spectroscopy can be used together with fluorescence techniques (28) and has been shown to provide complementary information in the case of 5-ALA (29).

However, Raman scattering is a weak phenomenon. Devices are very sensitive and, therefore, are affected by all other light sources and ambient light (30). This is of minor importance when working in a well-controlled laboratory environment. Here, physical measures can be taken to prevent the probe from light, for instance, working in darkness with light-shielding boxes. However, it is of utmost importance for the utilization of Raman spectroscopy in a clinical setting during surgery. In the operating room (OR), clinical needs define the light conditions. Furthermore, the sources of lightning greatly vary depending on the procedure and may include ceiling light, various screens, microscope illumination, invisible light from neuronavigation tools, and sunlight if the OR has windows. Here, we address the impact of spurious light on Raman spectra with the aim of providing practical guidelines for neurosurgeons using intraoperative Raman spectroscopy for *in situ* brain tumor analysis. Taking advantage of an experimental OR, we systematically investigated and characterized the effects of different light sources and disturbances on the Raman spectra of brain tissue and brain tumors.

## Materials and methods

### Tissue collection and processing

We used one sample each of human glioma (glioblastoma, *IDH*-wildtype, CNS WHO grade 4), human meningioma (meningothelial, CNS WHO grade 1) and rat brain. Human and animal tissue was fixed in 4% formalin. Raman spectroscopy measurements were directly performed on the formalin fixed bulk tissue.

### Ethics

Human brain tumor samples were collected during routine brain surgeries and patients gave informed consent beforehand. The study was approved by the ethics committee at the Technische Universität Dresden (EK: 323122008).

Animal tissue was obtained from rats in accordance with the guidelines of the Technische Universität Dresden, which are based on national laws that are in full agreement with the European Union directive on animal experimentation. With respect to the use of the 3R for animal protection, no animals were additionally killed for this study, but brain tissue from rats in other experiments was used (approved by the Regional Council Landesdirektion Sachsen, Germany, AZ: 25-5131-474-36). Rat brain explantation took place after regular killing and had no influence on the planning or execution of the animal experiments.

### Raman spectroscopy

Raman spectra were acquired with a handheld fiber-optic Raman probe (EmVision LLC., Loxahatchee, Florida, USA) in direct contact with the center of the tissue bulk. Depending on the tissue type used in the respective measurement series, the probe was either placed on tumor bulk without tumor borders (human glioma and human meningioma) or on normal tissue (rat brain). The fiber-optic handheld Raman probe was coupled to a continuous wave laser with a wavelength of 785 nm (Innovative Photonic Solutions, Monmouth Junction, New Jersey, USA) and a portable Raman system consisting of an EmVision HT spectrometer (EmVision LLC., Loxahatchee, Florida, USA) and an Andor DU420A-BR-DD CCD camera (Oxford Instruments, Tubney Woods, Abingdon, UK). The software Andor Solis (Oxford Instruments, Tubney Woods, Abingdon, UK) was used to control the CCD camera. A laser power of 80 mW on the sample was used and the total acquisition time was set to 2 s (40x accumulation with an integration time of 0.005 s).

### Data and Raman spectra analysis

Processing of the Raman spectral datasets was performed using MATLAB R2023a (The MathWorks, Inc., Natick, Massachusetts, USA) as described previously (31). Briefly, the background spectrum was subtracted from the raw spectrum followed by a baseline correction, artifact correction and normalization. The signal-to-noise-ratios (SNRs) of the Raman spectra were calculated in the range 1850 – 2000 cm^-1^. Data visualization and statistical tests were performed with GraphPad Prism 10 (GraphPad Software, Boston, Massachusetts, USA).

### Clinical environment

All the Raman spectral datasets were acquired in a fully equipped experimental OR. After the experimental environment was set up, illuminance was measured with a digital luxmeter (Goobay LX 101, Wentronic, Germany) for each measurement series, if applicable. The bulk tissue was placed on a petri dish lined with aluminum foil and positioned on the operating table where the head of the patient would be positioned in a clinical setting. Raman spectra were acquired with the fiber probe in direct contact with the tissue unless otherwise stated. The ambient light sources present in the experimental OR and considered in the analysis were: 1) microsurgical exoscope, 2) exoscope monitors, 3) infrared navigation system, 4) monitors of the OR, 5) ceiling lights and 6) operating (OP) lights. Specifications of the equipment used are provided in the **Supplementary Material** (**Supporting Table T1**). The measurements were performed at distance of 1 m as well as at a clinically reasonable distance to the ambient light source to the tip of the Raman probe, as indicated.

## Results

The Raman spectroscopic measurement provides a raw spectrum that is then mathematically processed to obtain an interpretable Raman spectrum with the characteristic pattern of Raman bands related to tissue constituents. The raw spectrum of biological tissue is composed of three components (**Supporting Supplementary Figure 1**): the Raman spectrum, the autofluorescence of the tissue which results from the excitation of endogenous fluorophores of the tissue, and the background (32). The Raman signal and autofluorescence are generated by the interaction of laser excitation with the tissue, while the background is present irrespective of excitation. This is caused by the dark current of the Raman CCD camera and any spurious light reaching the Raman detector, i.e. the sum of the near-infrared light from all sources entering the fiber probe during measurement, and spectrometer stray light. The background signal is usually acquired before the actual Raman measurement and subtracted from the raw spectrum during data processing (33).

**Figure 1:**
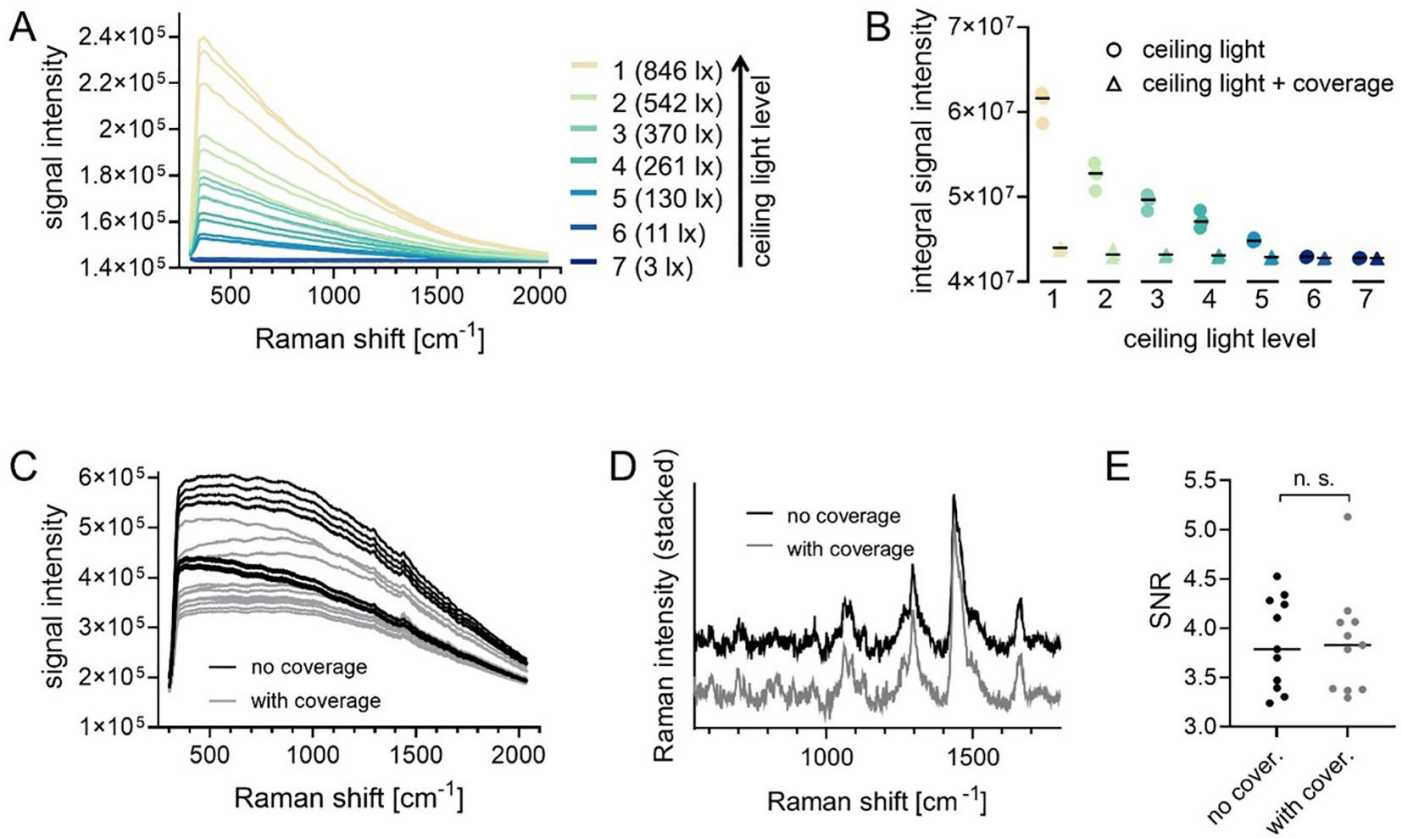
Effect of ceiling light on Raman spectroscopy of brain tissue. **(A)** Background spectra for ceiling light intensities from level 7 (lowest) to level 1 (highest) with their corresponding mean illuminance (lx). Background was acquired with the Raman probe in the measurement position (touching the rat brain tissue) with the laser switched off **(B)** Background integral intensities (400-1850 cm^-1^) for ceiling light intensities level 7 to level 1 without and with manual coverage. **(C)** Raw spectra of rat brain tissue acquired at light intensity level 1 without and with manual coverage. **(D)** Raman spectra extracted from the data shown in **(C)**. **(E)** Signal-to-noise-ratio (SNR) of Raman spectra obtained at light intensity level 1 without and with manual coverage, not significant (n.s.), Mann-Whitney test P= 0.6994.

Given that light conditions in the OR might impact clinical Raman spectroscopy by changing the background intensity, we investigated the influence of different ceiling light intensities using rat brain tissue samples. **Figure 1A** shows the background signal acquired in the experimental OR using the dimming levels 1-7 and there corresponding mean illuminance. In **Supporting Supplementary Figure 2**, we provide additional insight on the illuminance for different ceiling light levels. The Raman shift of 300 – 2000 cm^-1^ corresponds to near-infrared wavelengths of 804 – 931 nm. At low ceiling light intensities (levels 6 and 7), only minor background above the dark current was detected. With increasing ceiling light intensity, a gradual increase in background signal intensity was observed, importantly this did not lead to saturation of the Raman CCD. Shielding the Raman probe with hands was able to prevent the influence of the ceiling light on the signal (**Figure 1B**): With this manual coverage, which can be achieved by the surgeon, the background signals were reduced to intensities comparable to those of the ceiling light levels 6 and 7, which represented almost dark current signal intensities.

The raw spectra obtained from rat brain tissue under full ceiling light intensity without or with manual coverage are shown in **Figure 1C**. The intensities were generally higher than those of the background (compare **Figure 1A**). However, there is a large degree of heterogeneity between measurements and the intensity of the raw spectra is not necessarily lower when manual coverage is used. This suggests that the intensity of the raw spectrum is more related to the measurement position and tissue autofluorescence than to the ceiling light intensity (see **Supporting Supplementary Figure 3** for direct comparison of background and raw spectrum intensity).

Moreover, it becomes obvious that the intensity of the actual Raman bands of the brain tissue is very low compared with the intensity of the raw spectrum. Raman bands are hardly visible in the raw spectrum, and materials in the OR might have substantially stronger Raman signals (see **Supporting Supplementary Figure 4** for examples). This might also have implications for the quality of Raman spectra. The photon noise (i. e. the fluctuation in the number of photons detected by the CCD of the Raman system) follows a Poisson statistic (34): for N photons, the noise is proportional to N^1/2^. If the CCD pixel detects only the Raman photons, the stronger the signal (or the longer the acquisition time) the better the SNR. In this case the SNR improves as: N/N^1/2^=N^1/2^.

However, if the CCD also detects other sources of photons (such as autofluorescence and background light), the noise is determined by these other sources, which are usually much stronger than the weak Raman signal (see **Figure 1C** as an example). As a consequence, the Raman signal can be degraded by fluctuations associated with the spurious photon source; i.e., the SNR of the Raman spectrum decreases. However, in our setting, the ceiling light did not impair the spectral quality. Meaningful Raman spectra (**Figure 1D**) with similar SNRs (**Figure 1E**) could be extracted under both conditions.

**Figure 2:**
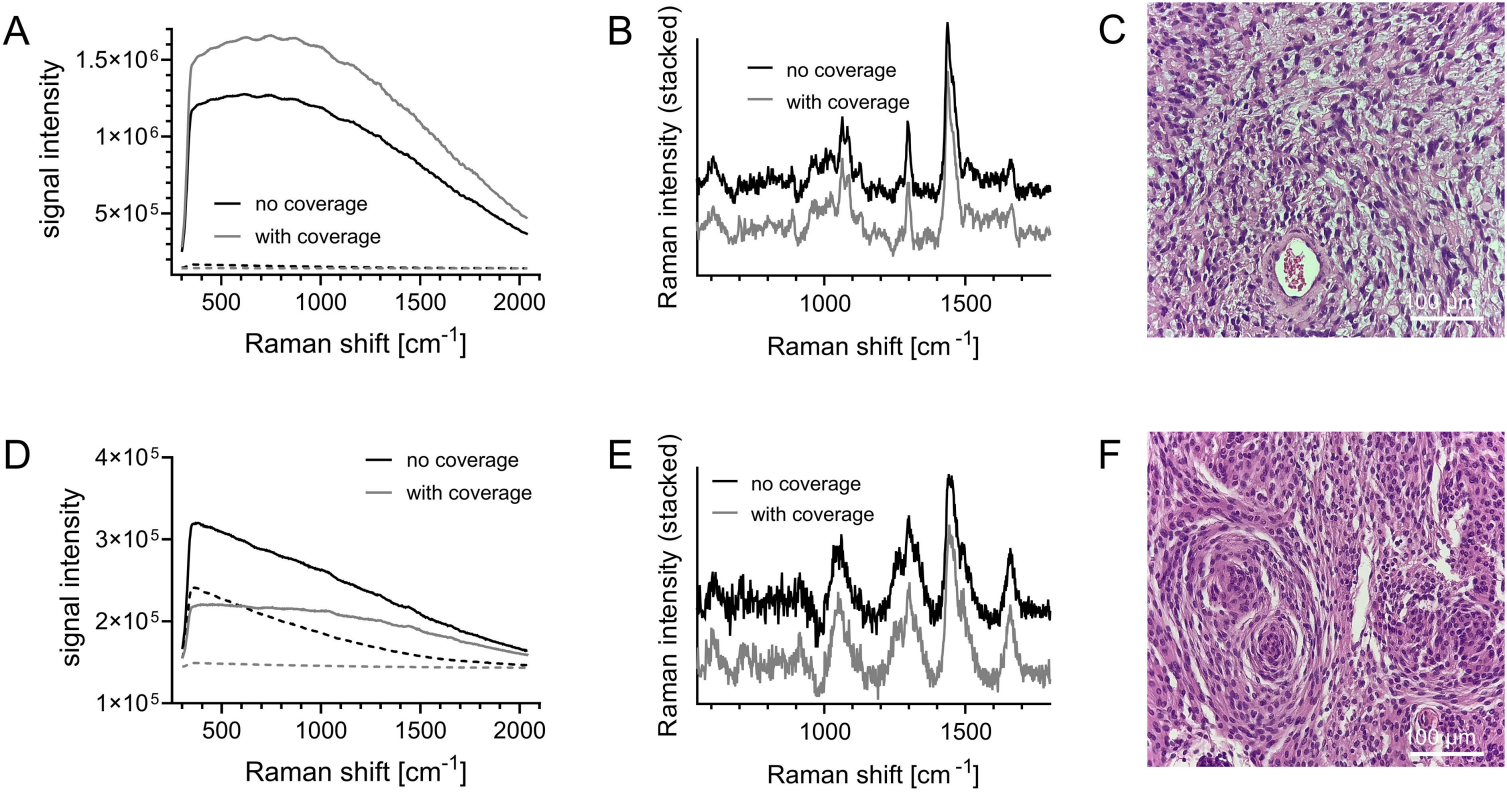
Effect of ceiling light on the Raman spectroscopy of brain tumors. Raman spectroscopy on samples of human glioma **(A, B)** and human meningioma **(D, E)** performed at ceiling light intensity level 1 without and with manual coverage. **(A, D)** solid line: raw spectra, dashed line: background. **(B, E)** Raman spectra. **(C, F)** are representative H&E stainings of the human glioma **(C)** and human meningioma **(F)**.

Previous research has shown that tissue specific autofluorescence is highly variable across different types of human brain tumors (16, 35–37). Therefore, we investigated human brain tumors with high and low autofluorescence. The intensity of the raw spectra of a glioma sample was one order of magnitude greater than the background values (**Figure 2A**), and the intensity of the raw spectrum obtained using manual coverage was greater than that obtained without manual coverage. This further supports the finding that the intensity of the background caused by ceiling lights is not critical for highly fluorescent (brain tumor) samples. Valid Raman spectra were also obtained for these conditions (**Figure 2B**). For meningioma, the intensity of the raw spectrum was lower than that shown for rat brain or glioma. Here, the raw spectrum is approximately twice the intensity of the background, and the raw spectra with and without coverage differ by the magnitude of the background (**Figure 2D**). Additionally, for this low fluorescent sample, valid Raman spectra with comparable SNRs were obtained with or without manual coverage (**Figure 2E**).

**Figure 3:**
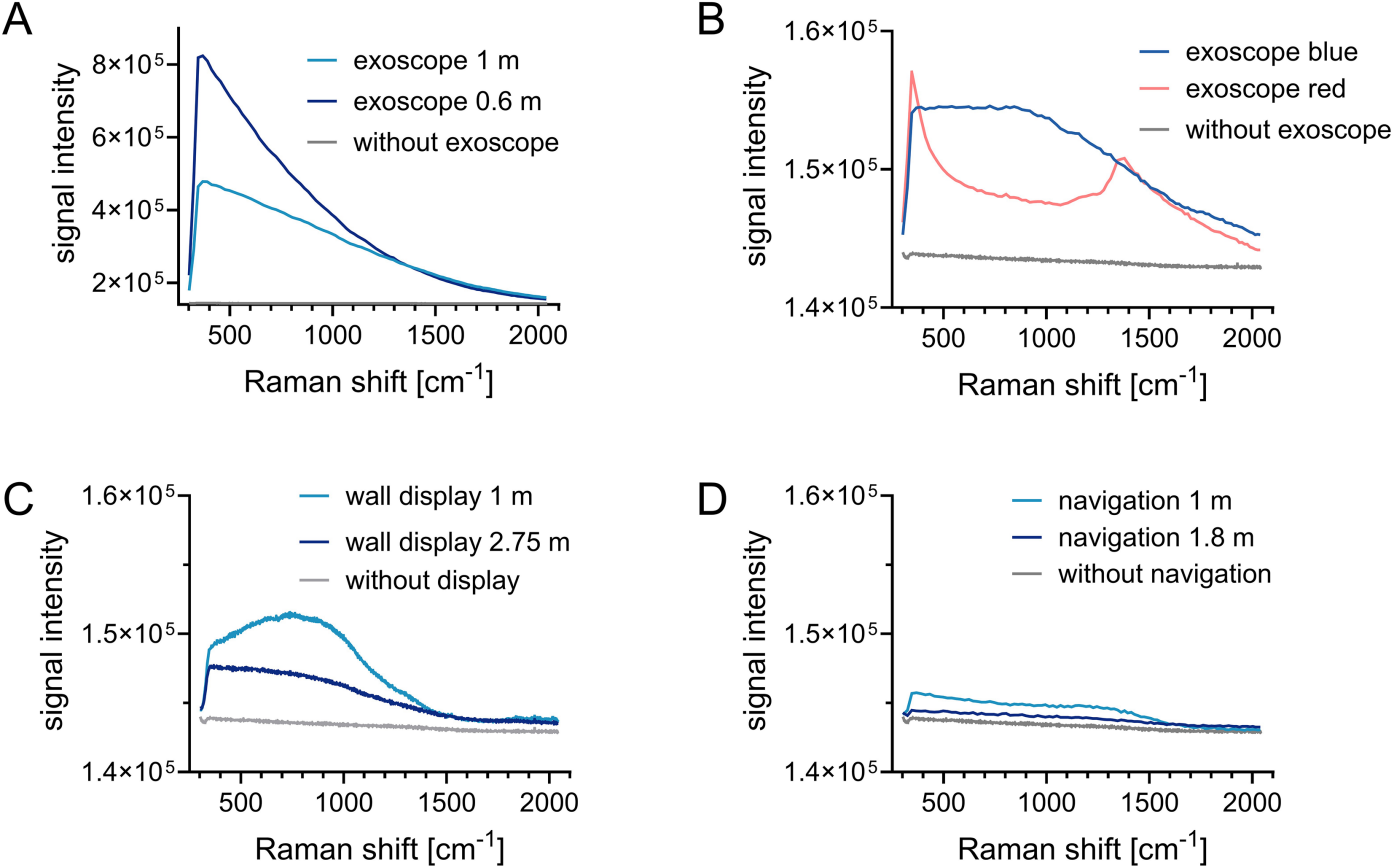
Signals caused by equipment in the operating room. All measurements were performed with the ceiling light intensity set to level 6, without tissue and the laser switched off. The background at ceiling light level 6 without further light source is shown for comparison (gray line). **(A)** Surgical exoscope in normal mode **(B)** Surgical exoscope in blue or red mode. **(C)** Display (LED screen), **(D)** Optical navigation system.

In addition to ceiling lights, screens of surgical equipment, optical instrumentation and tools might contribute to the background signal. The influence of OR-devices was investigated at two distances: at a uniform distance of 1 m, and at a distance relevant for clinical use which is specific to each device. On the basis of the data shown above, the following tests were performed with the ceiling lights switched on and set to level 6, thus providing sufficient illumination for operating and moving safely in the room but minimizing the influence on the background. Illumination with OP lights led immediately to a saturation of the CCD of the Raman system and is, therefore, not compatible with intraoperative Raman spectroscopy. The respective spectrum could be recorded only with manual coverage of the Raman probe (**Supporting Supplementary Figure 5**). The surgical exoscope induced a strong background signal (**Figure 3A**). When the blue or red mode was used, however, the background was much weaker and exhibited a different spectral shape (**Figure 3B**). The various screens of the OR induced a similar background signal in the range of 300 – 1400 cm^-1^, which diminished with increasing distance (wall display: **Figure 3C**; ceiling display: **Supporting Supplementary Figure 6**). The optical navigation system induced only a negligible background signal, even at a short distance of 1 m (**Figure 3D**). When the neurosurgical operating room was being screened, similar shapes of background signals were found for the screens. More interestingly, the optical navigation system used there caused a significant background signal (**Supporting Supplementary Figure 7A**), whereas the surgical microscope induced an intense background with defined bands, almost mimicking a Raman spectrum (**Supporting Supplementary Figure 7B**). Depending on the equipment and architecture, other sources of spectrally structured background might be fluorescent tubes or sunlight if there are windows in the OR (**Supporting Figures S7C, D**).

## Discussion

Disturbance of Raman spectroscopy by light is well recognized (33, 38) and previous reports of neurosurgical application of the technique have addressed this issue using different strategies. In most cases, special light regimes were established for Raman spectroscopic measurement. OP lights were turned off and operating room lights were used at low power (27), the light of the surgical microscope was switched off for the measurement (39) or the OR was darkened, but the OP lights, surgical microscope and optical neuronavigation were kept on (31). Moreover, physical measures, e.g. custom filters, have been developed and tested to avoid artifacts originating from the surgical microscope (40) while all datasets with artifacts were excluded from further analysis in other studies (41).

Our study confirms that several light sources in the OR might differentially impact measurements; thus, countermeasures are needed to ensure valid and relevant clinical Raman spectroscopy results. While OR-lights lead to saturation of the system, various light sources, including ceiling light at full intensity, increased only the background and were fully compatible with clinical Raman spectroscopy. Correct background measurements were sufficient for compensation of spectrally structured background (e.g., caused by optical neuronavigation) through data preprocessing. Optical neuronavigation has been previously shown to induce artifacts (41, 42). However, our data emphasize that this is not a general finding, and that the local situation needs to be screened and considered. This finding is further supported by a previous study investigating disturbances for clinical Raman spectroscopy, where neuronavigation was found to lead to saturation of the signal (42).

Brain tumors exhibit enormous inter- and intratumor heterogeneity in terms of autofluorescence patterns and intensity (16, 31, 37, 43). Our study indicates, that local tissue autofluorescence might dominate the intensity of the raw spectrum for brain tissue and several other brain tumor types, thereby representing a greater “disturbance” to the measurement than ceiling lights do, with potential implications for the SNR (34). When proper acquisition times were used, the presence of substantial autofluorescence of the glioma sample did affect the spectral quality or SNR. However, it is important to set the integration time of the Raman CCD within the dynamic range to avoid saturation and to verify whether and to what extent spurious photons impact the Raman SNR. For samples with low autofluorescence, the background accounts for a substantial portion of the raw spectrum. In those cases, the presence of an intensely structured background might largely impact the overall signal shape and intensity and interfere with some steps of data processing, such as artifact correction (31).

Notably, the results shown here are valid for near-infrared Raman spectroscopy only. If other wavelength ranges are used, the impact of spurious light on spectra may differ. This is particularly true when working in the visible range, where artifacts can be much stronger (32).

For successful clinical implementation of *in vivo* Raman spectroscopy, we suggest screening the OR for spectrally structured ambient light sources beforehand and shielding it from sunlight as well as intense infrared signals if it is compatible with clinical requirements. From a practical point of view, the most important thing is to avoid the saturation of the CCD of the Raman system (42), which might occur using long integration times, especially in the presence of spurious light or intense tissue autofluorescence. This effect might be worsened when exciting in the visible range (32). Second, the acquisition of a valid background signal is highly important for proper correction during data preprocessing. The background is acquired with the laser turned off, using the same acquisition parameters (integration time and number of accumulations) as those used for the actual Raman measurement. Moreover, the background needs to be acquired under the same external conditions used for the actual Raman measurement. For Raman measurements, the probe needs to be placed on the tissue, and eventually with manual coverage, all the instrumentation and personnel should be set and placed in position. The amount of collected ambient light depends not only on sample illumination, but also on the location of the Raman probe because the ambient light enters the probe after scattering and absorption processes within the tissue. Last but not least, one should be suspicious when strong, well-defined Raman bands are visible in the raw spectrum, because these bands are more likely to originate from OP material than from tissue (which typically has weak Raman bands) and might indicate mispositioning of the Raman probe.

## Conclusion

OR light sources do not impair the possibility of performing intraoperative Raman spectroscopy. Illumination levels compatible with normal OR functions can be used during Raman spectroscopy. However, before Raman spectra are acquired in a new OR environment, background screening of potentially disturbing light sources should be performed. This will enable several approaches to either avoid these spurious light sources or take them into account during data pre-processing. Finally, our data suggest, that the main issue affecting the quality of the Raman spectra of brain tissue is the intrinsic autofluorescence of the tissue.

## Data Availability

The raw data supporting the conclusions of this article will be made available by the authors, without undue reservation.
