# Supplementary material for: Relevance of light conditions for clinical in situ Raman spectroscopy
Source: Front Oncol. 2026 Jul 27;16:1850523. doi: 10.3389/fonc.2026.1850523 (PMC13453736; doi:10.3389/fonc.2026.1850523)
Supplement: Supplementary file 1 [file DataSheet1.docx]

# Supporting Information

**Supporting Table T1. Specifications of ambient light sources in the experimental OR.**

| **Ambient light source** | **Product** | **Company** |
| --- | --- | --- |
| Microsurgical exoscope | OrbEye OME-V200 | Olympus |
| Exoscope monitors | LCD-Monitor 31" Sony LMD-X310ST | Olympus |
| Infrared navigation system | CAS-One Navigationssystem, SN201 | CAScination |
| Wall monitors of the operating room | Wand-Monitor-42-2D-FHD-Touch-BrainLab; Wand-Monitor-49-2D-4K-Operion-rein-medical_BARCO | BrainLab |
| Ceiling monitors of the operating room | Decken-Monitor-27-2D-FHD-Touch-Clinio-rein-medical_BrainLab; Decken-Monitor-31-2D-4K-BARCO | BrainLab |
| Ceiling lights | CLEAN S-MP LED8000-940 L1250 LDO [STD] | Zumtobel |
| Operating lights | marLEDV10/V16 | KLS Martin |
|  |  |  |

**Supporting Figure S1. Schematic illustration of the different components of a raw spectrum**. The acquired raw spectrum consists of the background (caused by the dark current of the Raman CCD camera and room light reaching the probe during the measurement), the autofluorescence of the tissue which results from excitation of endogenous fluorophores of the tissue by the Raman laser and the Raman spectrum.

**Supporting Figure S2. Illuminance of the different ceiling light levels**. Points show single measurements for individual ceiling light levels. The horizontal line indicates the mean value (n=8).

**Supporting Figure S3. Comparison of background intensity and raw spectra intensity of brain tissue.** Background and raw spectra intensities acquired on rat brain tissue are shown for ceiling light intensities level 1 (highest) to level 7 (lowest) and without and with manual coverage. Integral intensities were calculated for the range of 400-1850 cm^-1^. Background was acquired with the Raman probe in measurement position (touching the rat brain tissue) with the laser switched off. Boxes indicate 25th to 75th percentiles, line at median, whiskers show range.

**Supporting Figure S4. Raman datasets of OR material.** Raw spectra and Raman spectra of selected material as indicated

**Supporting Figure S5. Background caused by OP lights (with manual coverage)**. Background was acquired with OP with lights switched on at a distance of 1.4 m. The ceiling light intensity set to level 6 and background was acquired with the Raman probe in measurement position (touching the tissue) and the laser switched off. The background without OP lights and ceiling light level 6 is shown for comparison (gray line).

**Supporting Figure S6. Background caused by ceiling displays**. The signal induced by the ceiling displays was acquired without sample, the laser switched off at distances of 1 m and 2.3 m. Background was investigated with the ceiling light intensity set to level 6; the background without display is shown for comparison (gray line).

**Supporting Figure S7. Background caused by instruments in neurosurgical operation room and other light sources**. **A:** Neuronavigation system (Curve Navigation, BrainLab SE). **B:** Surgical microscope (KINEVO 900, Carl Zeiss Meditech AG)). **C:** Sun light. **D:** fluorescent tube (Sylvania, Luxline plus, F18W/840 cool white).
